# Factorial structure of the Manchester Short Assessment of Quality of Life in patients with schizophrenia-spectrum disorders

**DOI:** 10.1007/s11136-019-02356-w

**Published:** 2019-11-12

**Authors:** Eleni Petkari, Domenico Giacco, Stefan Priebe

**Affiliations:** Unit for Social and Community Psychiatry (World Health Organization Collaborating Centre for Mental Health Services Development), Newham Centre for Mental Health, Queen Mary University of London, London, E13 8SP UK

**Keywords:** Subjective quality of life, MANSA, Factorial structure, Schizophrenia-spectrum disorders, EFA, CFA

## Abstract

**Purpose:**

Subjective quality of life is a central patient-reported outcome in schizophrenia-spectrum disorders. The Manchester Short Assessment of Quality of Life (MANSA) is an established and widely used instrument for its assessment. The present study is a secondary analysis of large schizophrenia studies and aims to establish the factorial structure of the MANSA with a rigorous two-step methodology.

**Methods:**

A sample of 3120 patients was randomly split into two datasets; the first includes two thirds of the patients and serves as the calibration sample (*N* = 2071) and the second includes one third of them and serves as the validation sample (*N* = 1049). We performed an exploratory factor analysis with the calibration sample followed by a confirmatory factor analysis with the validation sample.

**Results:**

Our results for both samples revealed a model with adequate fit comprising two factors. The first factor encompasses eight items measuring satisfaction with a variety of life and health-related aspects of quality of life, whereas the second consists of four items assessing satisfaction with living environment comprising living alone or with others, accommodation, family, and safety. These two factors correlate in a different way with socio-demographic characteristics such as age and living conditions.

**Conclusions:**

Future trials and service evaluation projects using the MANSA to measure quality of life should take into account that satisfaction with living environment may be distinct from satisfaction with other life and health-related aspects of quality of life.

## Introduction

Subjective quality of life (SQoL) is regarded as an important outcome in clinical practice and research [1–4] with patients with psychosis. One of the most widely used instruments to assess SQoL [1] is the Manchester Short Assessment of Quality of Life (MANSA [5]). The MANSA is based on Lehman’s [6] conceptualisation of quality of life and explores satisfaction with a number of life domains. It was created primarily for use in patients with schizophrenia-spectrum disorders and has been used in more than 700 studies. It is a brief, easily administered instrument that was developed as a shortened version of the Lancashire Quality of Life Profile (LQLP [4]) in order to reduce the length of the assessments and respondents’ fatigue. Thus, it can be easily included in research designs that involve extensive evaluations and also used in routine clinical practice.

An additional strength of this instrument is that the latent concept of quality of life measured is not specific to health-related issues. As such, it can be used to compare patients with psychosis to patients suffering from other types of mental illnesses, or to the general population. For these reasons, the MANSA offers an advantage compared to the more extensive and health-oriented quality of life instruments such as the WHO-QoL-bref [7] or the SF-36 [8]. Despite its extensive use, the factorial structure of the MANSA has not been established using rigorous statistical methods. A question remains as to whether the different MANSA items assess a unidimensional general appraisal of quality of life and life satisfaction [9], or if the MANSA assesses distinct latent constructs [10].

Previous attempts were made to answer this question, but they were based on incomplete versions of this instrument. Priebe et al. [11] examined psychometric properties of the DIALOG, a therapeutic intervention that includes eight out of the twelve MANSA SQoL items. Based on a sample of 271 patients, their aim was to test the feasibility of using the data extracted from the intervention as a valid SQoL patient report and thus the exploration of the complete MANSA structure was beyond their scope. Similarly, Eklund and Bäckström [12] measured the properties of only nine out of the twelve MANSA items as a part of an examination of SQoL determinants, using a sample of 161 patients. Both studies identified a two-factor structure of the MANSA. However, these studies would not provide useful information with regard to the factorial structure of the entire MANSA SQoL instrument, as in addition to not including all of the items, they were based on small and local samples and they did not perform a confirmatory factor analysis (CFA).

When validating an instrument, most analyses have the significant disadvantage of exploring and testing the model in only one sample. A proper methodology requires calibrating and validating the model in two different samples/sets. To address the above issues, a rigorous and systematic examination of the MANSA is required, including both an exploratory factor analysis (EFA) to develop a factorial model and a CFA to validate the findings in a different sample. Such systematic examination will allow us to provide recommendations for an accurate use of the instrument in clinical practice and research.

## Materials and methods

### Procedure

For the purpose of the present analysis, we merged the data of nine different studies that assessed SQoL using the MANSA in patients with schizophrenia-spectrum disorders (ICD-10: F20-F29 [13]). Patients were above 18 years old, had the capacity to provide informed consent, and had sufficient command of the language of the country where they were assessed. Those suffering from any type of organic brain disorders or cognitive impairment were excluded. Overall, the merged database included *N* = 3120 patients. Details of the included studies can be seen in Table 1. When data on SQoL were available for more than one time point, we opted to include only baseline scores, to obtain as much data as possible from each study.

**Table 1:** Details of studies included in the analysis

Study	Authors	Study type	Aim	*N* patients included	Patients type	Country sites of included patients
EUNOMIA	Kallert et al. [30]	Prospective observational	Examine the coercive measures used in psychiatric treatment, together with influencing factors and outcomes of coerciveness	759	Inpatients	Germany Poland Sweden Czech Republic Slovakia Lithuania Israel
InvolvE	Priebe et al. [31]	Prospective observational	Examine the coercive measures used in psychiatric treatment, together with influencing factors and outcomes of coerciveness	369	Inpatients	UK
VOLUME	Toner et al. [32]	Survey	Study the preferences for befriending of psychotic patients	151	Outpatients	UK
VOLUME RCT	Priebe et al. [33]	Randomised controlled trial	Study the effectiveness of volunteer support to psychotic patients with limited social activities	123	Outpatients	UK
COFI	Giacco et al. [34]	Prospective natural experiment	Explore whether personal continuity or specialisation of psychiatrists is associated with patient outcomes one year after hospitalisation	739	Inpatients	UK Germany Italy Poland Belgium
DIALOG	Priebe et al. [35]	Clustered randomised controlled trial	To investigate the impact of a new intervention regularly used in routine clinician–patient meetings on quality of life, unmet needs for care, and treatment satisfaction	507	Outpatients	UK Germany Spain Switzerland Sweden Netherlands
EDEN	Kallert et al. [36]	Randomised controlled trial	To compare acute day care with conventional inpatient treatment	255	Inpatients	UK Germany Poland Czech Republic Slovakia
STAR	McGuire-Snieckus et al. [37]	Prospective observational	To develop an instrument assessing the clinician–patient relationship	77	Outpatients	UK Sweden
Exploratory models	McCabe and Priebe [38]	Cross-sectional observational	To explore explanatory models of mental illness and their associations with clinical and social variables	140	Outpatients	UK

### Measures

The subjective components of the MANSA scale encompasses 12 items that measure satisfaction with life as a whole, job or being unemployed, financial situation, number and quality of friendships, sex life, leisure activities, accommodation, personal safety, people living with or living alone, family relationships, physical health, and mental health. Satisfaction is measured using a 7-point Likert scale, from 1: could not be worse to 7: could not be better. The instrument is clinician-administered or self-rated and it takes up to 15 min to be completed. It has been found to have satisfactory psychometric properties in terms of concurrent validity, overall reliability [6], and internal consistency [14].

### Statistical analyses

The analyses were carried out using SPSS v.24. Before proceeding, we inspected the data for normality, outliers, and missing values. Visual inspection of the histograms revealed a normal distribution of the data and boxplot inspection showed no outliers. Regarding missing data, this was less than 4% across the 12 MANSA items (with the exception of item 5-Satisfaction with sex life, where the percentage was 12%). First, we randomly split the sample (*N* = 3120) and created two separate datasets, the first including two thirds of the initial sample (*N* = 2071) and serving as the calibration sample and the second including one third of the initial sample (*N* = 1049) and serving as the validation sample. Potential differences between the two samples were tested by using *T*-test or Chi square analyses. In order to validate the MANSA structure, we performed an EFA using a maximum likelihood estimation process with the calibration sample and a CFA with maximum likelihood with the validation sample, so as to corroborate the solution offered by the first analysis.

For the EFA, we applied oblique rotation with Kaiser normalisation instead of varimax, so as to allow for possible correlations between the factors [15]. Pairwise deletion was used to handle missing data [16]. In order to determine the number of significant Eigenvalues to be extracted from the data, we ran a parallel analysis with Montecarlo simulation [17]. Finally, we used the JASP software to calculate the omega coefficient (ω) for each of the obtained factors to check their reliability.

We also performed sensitivity analyses by repeating the EFA, first excluding the first item (satisfaction with life as whole), to examine whether the factorial structure is influenced by this item due to its generic nature, and second by omitting item 5 (satisfaction with sex life), due to the amount of missing data, to examine whether this would affect the final factorial solution. Also, we applied a Chi square difference test, to compare the fit of the proposed solution with that of a model where all items were loaded in a single factor.

For the CFA, we used AMOS (Analyses of Moment Structures) with the validation sample. Although using the Chi square statistical test is a common practice to determine the model’s goodness of fit when the *p* value is < .05, this is not recommended for large datasets, as it is influenced by the sample size [18]. To account for such drawback, based on Hu and Bentler’s [19] recommendations for avoiding Types I and II error, we used a combination of indexes to estimate the goodness of fit of our model. Specifically, we used the Root Mean Square Error of Approximation (RMSEA) that assesses the errors in fitting the data to the covariance matrix, with values below .05 representing an excellent fit and narrow confidence intervals from .00 to .08 indicating a good model fit [20]. We also considered the Comparative Fit Index (CFI) that provides a comparison of the hypothesised model to an unfit model, delivering a measure of complete variation of the data and showing an adequate fit when the values are > .95 [19] and acceptable when > .90 [21].

Finally, to check whether the same general specification for the model holds across groups, we examined the measurement invariance for gender (male, female), service setting (inpatients, outpatients), and living situation (alone, other). We did this by pooling the general fit across groups and checking the configural, metric (factor loadings are equal across groups), scalar (the observed scores are related to the latent scores regardless of the group), and strict invariance (the residuals are equal showing the same amount of error across groups), across categories for each of these variables. Then, we performed additional analyses to assess whether the proposed factors showed diverse associations with those variables, as an estimation of their distinctive nature. Concretely, we used three independent samples *T*-test to test their relationship with gender, service setting, and living situation. We also carried out a Pearson correlation analysis to test their relationships with age.

## Results

### Sample characteristics

The socio-demographic and clinical characteristics of the total sample can be seen at Table 2. The comparative analyses between the calibration and the validation sample revealed the absence of statistically significant differences in gender [*χ*^*2*^ (1) = 1.110, *p *= .292], age [*t* (3010) = .186, *p *= .852], education [*χ*^*2*^ (3) = 1.836, *p *= .607], marital status [*χ*^*2*^ (5) = 3.040, *p *= .694], living situation [*χ*^*2*^ (1) = 2.887, *p *= .089], employment [*χ*^*2*^ (6) = 4.425, *p *= .619], study country [*χ*^*2*^ (12) = 13.029, *p *= .367], service setting [*χ*^*2*^ (1) = 0.43, *p *= .836], or number of previous admissions [*t* (1458) = − .852, *p *= .852].

**Table 2:** Psychosocial and clinical characteristics of the sample (*N* = 3120)

Factor	*N* (%)	Mean	*SD*
Gender (*N* = 3118)			
Male	1901 (61)		
Female	1217 (39)		
Age (*N* = 3012)		39.37	11.65
Marital status (*N* = 2602)			
Single/unmarried	1656 (58.7)		
Married/partnership	534 (18.9)		
Separated/divorced	369 (13.1)		
Widowed	43 (1.5)		
Education (*N* = 1045)			
Primary or less	208 (19.8)		
Secondary	430 (40.9)		
Tertiary	358 (34.0)		
Other	49 (4.7)		
Age when leaving education (*N* = 1957)			
< 17	601 (30.7)		
17–18	493 (25.2)		
19–22	468 (23.9)		
23+	395 (20.2)		
Living situation (*N* = 2664)			
Alone	1349 (50.6)		
Other	1315 (49.4)		
Employment (*N* = 3100)			
Employed	431 (13.9)		
Unemployed	1519 (49)		
Retired/household	800 (25.8)		
Student	120 (3.8)		
Sheltered/volunteer/other	163 (5.3)		
Study country (*N* = 3120)			
UK	1288 (41.3)		
Germany	291 (9.3)		
Italy	129 (4.1)		
Spain	88 (2.8)		
Poland	402 (12.9)		
Sweden	107 (3.4)		
Czech Republic	160 (5.1)		
Slovakia	220 (7.1)		
Lithuania	152 (4.9)		
Israel	36 (1.2)		
Belgium	71 (2.3)		
Netherlands	99 (3.2)		
Study setting (*N* = 3120)			
Inpatient	2122 (68)		
Outpatient	998 (32)		
Number of admissions (*N* = 1460)		2.77	8.06
Number of involuntary admissions (*N* = 687)		4.67	6.70

The Means and Standard Deviations for the MANSA items across the calibration and validation samples can be seen at Table 3. In both samples, patients seemed to have the highest scores for items evaluating satisfaction with accommodation, people living with, safety, and family (items 7, 8, 9, and 10) and the lowest for items assessing finance and sex life (items 3 and 5).

**Table 3:** Means and SDs of the MANSA items

MANSA items	Calibration sample (*N* = 2071)		Validation sample (*N* = 1049)	
	Mean	*SD*	Mean	*SD*
1. Life as a whole	4.35	1.71	4.47	1.71
2. Job/unemployment	4.10	1.82	4.14	1.81
3. Financial situation	3.96	1.81	3.92	1.79
4. Friendships	4.52	1.74	4.53	1.71
5. Sex life	3.85	1.88	3.91	1.86
6. Leisure activities	4.48	1.72	4.49	1.69
7. Accommodation	4.87	1.79	4.80	1.79
8. Living situation	4.85	1.72	4.88	1.67
9. Personal safety	4.73	1.71	4.72	1.70
10. Family	4.83	1.72	4.79	1.70
11. Physical health	4.62	1.68	4.64	1.62
12. Mental health	4.41	1.76	4.40	1.71

### Results of the exploratory factor analysis (calibration sample)

The correlation matrix showed that all the factors correlated with each other. The parallel analyses indicated the existence of two factors with significant Eigenvalues. The first factor (satisfaction with life and health-related aspects) had an Eigenvalue of 4.05 and included items assessing satisfaction with life as a whole, job or being unemployed, financial situation, number and quality of friendships, sex life, leisure activities, physical and mental health (1, 2, 3, 4, 5, 6, 11, and 12), and the second factor (satisfaction with quality environment) had an Eigenvalue of 1.12, and included items assessing satisfaction with accommodation, personal safety, people living with or living alone, and family relationships (7, 8, 9, and 10). The correlation between the two factors was *r *= .62. The load coefficients per item (in bold) together with the reliability of the two factors can be seen at Table 4.

**Table 4:** Factor components and item loading according to the EFA of the MANSA (*N* = 2071)

MANSA items	Factor loadings	
	Factor 1 Life and health-related aspects	Factor 2 Quality of environment
Coefficient omega (ω)	.777	.678
12. Mental health	**.673**	-.119
6. Leisure activities	**.643**	-.079
1. Life as a whole	**.626**	.127
11. Physical health	**.516**	.008
4. Number and quality of friendships	**.505**	.082
2. Job/Being unemployed	**.421**	.059
3. Financial situation	**.360**	.210
5. Sex life	**.319**	.175
8. People living with or living alone	-.092	**.736**
10. Relationships with family	.123	**.509**
7. Accommodation	.114	**.470**
9. Personal safety	.274	**.305**

When repeating the analyses after excluding item 1 (satisfaction with life as a whole), the structure remained the same. However, the reliability for Factor 1 (satisfaction with life and health-related aspects) was lower (Coefficient Omega = .730). When doing the same by excluding item 5 (satisfaction with sex life), the structure did not change either but the reliability of Factor 1 (satisfaction with life and health-related aspects) was lower when excluding this item (Coefficient Omega = .767). Therefore, we opted for adopting the two-factor solution including all the MANSA items.

### Results of the confirmatory factor analysis (validation sample)

**Fig. 1:**
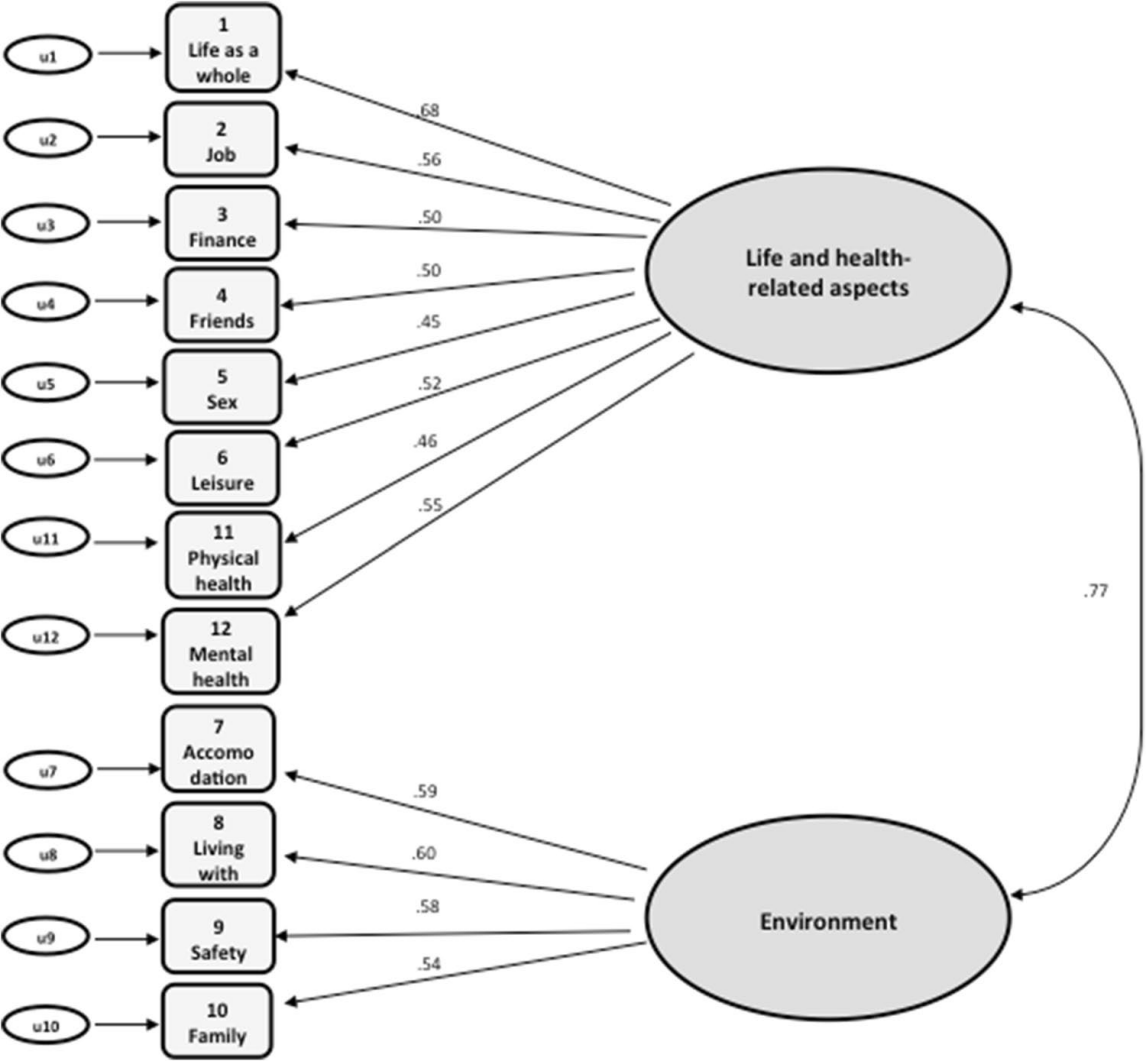
Standardised parameters of the MANSA item loadings to two factors

The CFA confirmed the solution provided by the EFA. The RMSEA fit index was higher than .05 but had a narrow confidence interval revealing an acceptable goodness of fit for the model [RMSEA = .067; 95% CI (.060, .075)], similar to the CFI (= .90). The standardised item loadings for each factor are seen at Fig. 1. All loadings were significant and exceeded .40, ranging from .45 to .68. Finally, the inter-factor correlation was *r *= .77, which is below the threshold of .80, confirming the existence of distinct multidimensional factors comprised under the latent SQoL construct.

The analysis excluding item 1(satisfaction with life as a whole) showed similar but slightly poorer fit: [RMSEA = .068; 95% CI (.060, .077)] whereas the CFI was marginally below the acceptable threshold (CFI = .89). The results when excluding item 5 (satisfaction with sex life) were similar [RMSEA = .072; 95% CI (.064, .081) and (CFI = .89)]. In addition, the comparison of the two-factor solution with a single-factor model encompassing all the items revealed no significant differences [*χ*^*2*^*diff* (1) = 1.596; *p *= .10), indicating that the single-factor solution may be used as an alternative. However, the latter showed poorer fit [RMSEA = .078; 95% CI (.071, .085) and (CFI = .86)].

Measurement invariance tests for gender revealed that across males and females, there was configural invariance with excellent fit [RMSEA = .048; 95% CI (.042, .085); CFI = .90], as well as good metric [RMSEA = .046; 95% CI (.041, .051); CFI = .89; *χ*^*2*^(10) = 15.144; *p *= .127], scalar [RMSEA = .045; 95% CI (.040, .050); CFI = .88; *χ*^*2*^(3) = .440; *p *= .932], and strict invariance [RMSEA = .043; 95% CI (.039, .048); CFI = .88; *χ*^*2*^(12) = 11.140; *p *= .517] . The same was revealed for living situation: [RMSEA = .050; 95% CI (.044, .056); CFI = .88], metric[RMSEA = .048; 95% CI (.042, .054); CFI = .88; *χ*^*2*^(8) = 7.931; *p *= .440], scalar [RMSEA = .049; 95% CI (.044, .055); CFI = .87; *χ*^*2*^(3) = 4.216; *p *= .239], and strict invariance [RMSEA = .047; 95% CI (.042, .052); CFI = .86; *χ*^*2*^(12) = 20.511; *p *= .058]. Lastly, the model held across service settings with good fit [RMSEA = .050; 95% CI (.044, .055); CFI = .89], showing metric invariance with no significant differences between inpatients and outpatients in factor loadings [RMSEA = .047; 95% CI (.042, .052); CFI = .89; *χ*^*2*^(8) = 6.231; *p *= .621]. However, there was evidence for scalar non-invariance [RMSEA = .047; 95% CI (.042, .052); CFI = .87; *χ*^*2*^(3) = 9.281; *p *= .026]; thus, further invariance testing was stopped and potential differences in Factors 1 and 2 for patient type could not be explored further.

### Relationships of factor 1 (life and health-related aspects) and 2 (quality of living environment) with gender, service setting, age, and living situation

The *T*-test analyses showed that men and women were not statistically different across Factors 1(satisfaction with life and health-related aspects) [*t*(3115) = .053; *p *= .958] and 2(satisfaction with quality environment) [*t*(2463) = − 1.276; *p *= .202]. Age showed a weak positive correlation with Factor 1 (*r *= .036; *p* < .05) and no correlation with Factor 2. Finally, Factor 1 was not found to be different between people living alone and those having another living situation such as family, friends, or sheltered housing [*t*(2662) = − 1.038; *p *= .299] but Factor 2 was clearly different between the two living situations [*t*(2661) = − 5.100; *p *< .001], with people living alone showing less satisfaction (*M *= 4.63; *SD *= 1.27) than people in other living situations (*M *= 4.88; *SD *= 1.23).

## Discussion

### Main findings

Our results provide evidence that SQoL as measured by the MANSA comprises two distinct but correlated factors. The first factor incorporates several indicators of life and health, such as satisfaction with life as a whole, physical and mental health, leisure activities and friends, job/unemployment and financial situation, and sex life. The second factor encompasses satisfaction with family, accommodation, safety, and their living situation (whether they are living with someone or alone). This factor expresses a latent variable related to satisfaction with quality of living environment, which may be regarded as a separate aspect of SQoL. The model has an adequate fit and includes all the MANSA items in its two-factor structure. Sensitivity analyses performed by excluding certain items did not considerably change the structure, further supporting the robustness of the model. Additionally, though the model does not show better fit when compared to a single-factor model, it provides a more in-depth examination of quality of life components, and thus, its use can be considered more advantageous in research and clinical practice. The confirmation of measurement invariance followed by the distinct associations of the two factors with socio-demographic variables added evidence for their discrete nature and distinctive rating value for exploring associations of SQoL with other variables.

### Strengths and weaknesses

This is the first systematic examination of the MANSA factorial structure. The analyses were based on a large sample of patients with schizophrenia-spectrum disorders, from different countries. This allowed us not only to have appropriate statistical power, but also to include variation in our sample related to different contexts and cultures. Additionally, we used a methodologically sound procedure to cross-check the accuracy of the proposed solution by randomly splitting the sample and by implementing two separate analytical procedures. Sensitivity analyses were designed and carried out to further check the robustness of the findings.

However, the present study also has some limitations. First, the reliability indexes for both factors were acceptable but not high. Although these were not high, they are consistent with the reliability indexes of the SQoL component of the MANSA (α = .74) as reported in its initial validation [5]. Similarly, the goodness of fit indexes for the CFA revealed acceptable but not excellent fit of the model, though when excluding items as a means of addressing this, the goodness of fit did not change dramatically. Second, despite the fact that for all the other items the percentage of missing values was under 4%, the item assessing satisfaction with sex life had a higher percentage of missing data (12%), perhaps reflecting the patients’ or the clinicians’ reluctance to speak about this issue [22]. Satisfaction with sex life had the lowest loading to the first SQoL factor. Although the sensitivity analyses suggested that the item can be maintained without significant reliability changes, perhaps sex life demands further exploration as a separate domain of quality of life, particularly considering that its scores are the lowest among the MANSA items, which is in line with previous [23, 24]. Third, the study samples included people at different stages of their illness. It is known that SQoL ratings can vary between patients experiencing their first psychotic episode and those with a longer duration of illness [25]. However, we could not test whether this influenced our results because reliable information on illness duration was not available across all of the included studies. Similarly, the fact that the MANSA can be both clinician and self-report administered may have influenced the patients’ SQoL-reported ratings, but data on the administration form were not available across studies. Nevertheless, evidence from such comparisons of other instruments used in the routine clinical practice revealed no significant influence of the administration form on the reported outcomes [26].

### Comparison with previous literature

Previous analyses of the MANSA items also supported a two-factor solution [11, 12]; however, the composition of the factors was different to the one proposed by the present results, perhaps due to the lack of inclusion of all items and small sample sizes in the former studies. Eklund and Backstrom [12] did find that satisfaction with family, personal safety, and accommodation clustered together, whilst Priebe et al. [11] found that satisfaction with personal safety was included in a different factor, along with satisfaction with mental and physical health. Yet, the joint evidence from these three studies strongly supports a two-factor model for the MANSA. Our study, in consideration of its methodological strengths (higher statistical power, inclusion of all items, and analysis of data from international samples), might be regarded as better suited than previous ones to identify the nature of the specific items included within each factor.

### Implications

A two-factor structure of the MANSA may provide a hypothesis for explaining the frequently replicated finding that patients with schizophrenia report high levels of SQoL despite their often disadvantaged living conditions, a phenomenon known as the “disability paradox” [27]. Indeed, previous studies have consistently reported weak associations between objective indicators and SQoL [28, 29]. The present analysis specifies those findings further. Specifically, the latent domain related to quality of living environment appears to be correlated to objective living conditions, whilst the other factor (satisfaction with life and health) does not. It may be the case that the satisfaction with life and health domain is more dependent on a general appraisal tendency, which is not influenced by objective life conditions, whilst satisfaction with living environment is more directly affected by real-life conditions. Further studies should confirm whether the different patterns of correlations between the two factors and objective life conditions can be replicated, using the proposed two-factor structure of the MANSA Also, future research should explore the feasibility of a bi-factor solution, testing whether SQoL as measured by the MANSA could in fact represent an underlying construct with two domain-specific factors.

A two-factor model of quality of life may help to evaluate interventions of different types. For example, interventions that are principally aimed to improve satisfaction with health or personal life domains might be better assessed using a subscale reflecting the items included in our factor related to satisfaction with personal life and health. Social interventions targeting housing and neighbourhoods may, instead, benefit from a more specific and sensitive subscale to measure their effects, which may be represented by our factor related to satisfaction with living environment. Being aware of these two latent constructs within the MANSA can therefore be used to tailor how this instrument is used in evaluation protocols of routinely provided mental health care, or research studies of novel interventions.
